# Predatory Strategies of *Myxococcus xanthus*: Prey Susceptibility to OMVs and Moonlighting Enzymes

**DOI:** 10.3390/microorganisms11040874

**Published:** 2023-03-29

**Authors:** Allison S. Zwarycz, Thomas Page, Gabriela Nikolova, Emily J. Radford, David E. Whitworth

**Affiliations:** Department of Life Sciences, Aberystwyth University, Aberystwyth SY23 3DD, UK

**Keywords:** extracellular vesicle, glyceraldehyde-3-phosphate dehydrogenase, membrane fusion, outer membrane vesicle, phosphoglycerate kinase, predation

## Abstract

Predatory outer membrane vesicles (OMVs) secreted by myxobacteria fuse readily with the outer membranes of Gram-negative bacteria, introducing toxic cargo into their prey. Here we used a strain of the myxobacterium *Myxococcus xanthus* that produces fluorescent OMVs to assay the uptake of OMVs by a panel of Gram-negative bacteria. *M. xanthus* strains took up significantly less OMV material than the tested prey strains, suggesting that re-fusion of OMVs with producing organisms is somehow inhibited. The OMV killing activity against different prey correlated strongly with the predatory activity of myxobacterial cells, however, there was no correlation between OMV killing activity and their propensity to fuse with different prey. It has previously been proposed that *M. xanthus* GAPDH stimulates the predatory activity of OMVs by enhancing OMV fusion with prey cells. Therefore, we expressed and purified active fusion proteins of *M. xanthus* glyceraldehyde-3-phosphate dehydrogenase and phosphoglycerate kinase (GAPDH and PGK; moonlighting enzymes with additional activities beyond their roles in glycolysis/gluconeogenesis) to investigate any involvement in OMV-mediated predation. Neither GAPDH nor PGK caused lysis of prey cells or enhanced OMV-mediated lysis of prey cells. However, both enzymes were found to inhibit the growth of *Escherichia coli*, even in the absence of OMVs. Our results suggest that fusion efficiency is not a determinant of prey killing, but instead resistance to the cargo of OMVs and co-secreted enzymes dictates whether organisms can be preyed upon by myxobacteria.

## 1. Introduction

Predation is a fundamental biological phenomenon that shapes the composition of ecosystems and drives the evolution of ecosystem members. Biological control exploits natural predation by directing it towards non-natural prey species that are problematic for humans. While historically considering macrofauna and megafauna, this concept can be extended to microbial predators, which have the potential to control problematic microbes such as bacterial pathogens. To this end, diverse microbial predators have been bio-prospected and their predatory activities characterised [1].

The bacterial genus *Herpetosiphon* and the order Myxococcales (myxobacteria) are commonly described as ‘wolf-pack’ predators [1,2]. They secrete lytic toxins into the public commons, killing a broad range of microbial prey [3,4,5]. Although multiple wolf-pack predators in the same vicinity might contribute to killing the same prey, whether this behaviour is truly cooperative and akin to predation by wolf-packs is uncertain [6]. Of the wolf-pack predators, myxobacteria are currently the best-characterised [7].

In the model myxobacterium *Myxococcus xanthus*, the presence of prey and/or prey secretions promotes predatory behaviours, including regulated changes in motility [8,9]. Contact-dependent mechanisms involved in prey killing have recently been described [10,11]; however, prey killing can also be mediated by the constitutive secretion of toxins, both within outer membrane vesicles (OMVs) and in the soluble extracellular medium [12,13]. OMVs are a type of bacterial extracellular vesicle (BEV), and the nomenclature of BEVs is evolving with the discovery of different mechanisms of release [14]. In this paper we will refer to OMVs for consistency with previous studies of *M. xanthus*. Several studies have shown that *M. xanthus* OMV cargo proteins are enriched for diverse digestive hydrolases and proteins of unknown function [15,16,17,18]. OMVs maintain the concentration of their cargo proteins at a distance, but also serve to reduce the transport of toxins away from the producing cell, potentially preventing exploitation by competing cells [6,19].

OMVs deliver toxic cargo proteins and metabolites to Gram-negative prey cells by fusing with their outer membranes, and to Gram-positive bacteria by adhering to their outer surface and lysing [20]. This makes OMVs of interest to biotechnology for two reasons—as potential sources of novel antimicrobials, and as loadable delivery devices [20,21,22,23]. The killing of Gram-negative bacteria by *M. xanthus* OMVs requires the OMVs to be topologically intact and appears to be promoted by the fusogenic enzyme GAPDH (glyceraldehyde-3-phosphate dehydrogenase), which is secreted by *M. xanthus* within and alongside OMVs [12]. It is not clear to what extent OMVs are responsible for the predatory activity of myxobacterial cells, but it seems likely that they are primary determinants of contact-independent predatory activity, either directly or indirectly, as even the composition of the soluble secreted proteome appears to be largely due to release of material from lysed OMVs [12].

The prey range of *M. xanthus* is very broad, with strains able to effectively prey upon bacteria (Gram-negative and Gram-positive) and fungi. However, the prey range is also very patchy, with strain-to-strain differences in prey susceptibility to predation [3,4]. It is thought that this patchiness reflects the acquisition/loss of predatory determinants by the predator and resistance genes by the prey, as the prey range does not correlate with the phylogeny of the predator or prey [4]. Consistent with this interpretation, the myxobacteria have extremely open pan-genomes, with each strain possessing a largely unique accessory genome [18,24,25], hampering genome-wide association studies attempting to identify predation genes [26,27]. The variability of the gene sets between strains is also mirrored in the OMV proteome, which despite some commonality, differs substantially between strains of *M. xanthus* [18].

Some prey species are more resistant than others to attack by particular myxobacterial strains, and even within a species, different strains of prey can exhibit markedly different predation susceptibilities [4]. The same is also true for attack by secreted OMVs (our unpublished observations). Resistance to attack by OMVs could be a consequence of the resistance to the toxic cargo of OMVs, or by inhibition of OMV fusion to the outer membrane—preventing delivery of potentially toxic cargo proteins. Here, to investigate the hypothesis that predatory activity correlates with the fusion of myxobacterial OMVs with prey cells, we assayed the uptake of myxobacterial OMVs by a variety of Gram-negative prey organisms. We also investigated the relationships between the prey organisms’ resistance to myxobacterial OMVs/cells and their propensity to fuse with myxobacterial OMVs. 6His-fusion proteins of *M. xanthus* GAPDH and PGK were expressed and purified, which allowed us to reconstitute the OMV-mediated predatory regimes in vitro, providing novel insights into the roles of GAPDH, PGK and OMVs in myxobacterial predation.

## 2. Materials and Methods

### 2.1. Strains and Growth Conditions

Myxobacteria strains. CA005, CA018 and AB056 are wild-type isolates of *M. xanthus* with distinctive prey ranges from one another [4]. *M. xanthus* strain EH715 was created by Egbert Hoiczyk and Jeffery So [28], and expresses mCherry under the control of a vanillate-inducible promoter. The *M. xanthus* strains were grown at 30 °C in a DCY medium (2% w/v casitone, 0.2% w/v yeast extract, 8 mM MgSO_4_, 10 mM Tris, pH 7.8, solidified with 1.5% w/v agar). To induce the expression of mCherry, EH715 was grown in DCY supplemented with 200 µM vanillate and fluorescent cells imaged using excitation and emission wavelengths of 560 nm and 610 nm, respectively, using a Leica SP8 Super Resolution Laser Confocal Microscope.

Prey strains. The following strains were used as prey organisms in this study: *Escherichia coli* TOP10, *Xanthomonas campestris* pv. campestris NCPPB 528, *Pseudomonas syringae* pv. tomato DC3000, *Pantoea agglomerans* NCPPB 138, *Pectobacterium atrosepticum* NCPPB 1269, and *Rhizobium radiobacter* NCPPB 2655. Prey were grown in LB (0.5% *w*/*v* tryptone, 0.25% *w/v* yeast extract, 0.5% *w/v* NaCl, pH 7, solidified with 1.5% *w/v* agar) and incubated at 30 °C.

### 2.2. OMV Production and Characterisation

OMV purification was performed by both ultracentrifugation and size exclusion chromatography. To generate fluorescent OMVs, 100 mL cultures of EH715 were supplemented with vanillate to a final concentration of 200 µM, 24 h prior to harvesting (late-exponential phase). After harvesting, the cells were removed from the cultures as described previously [18]. The OMVs were then purified from portions of the resulting supernatants by ultracentrifugation, as described by [12], and by size exclusion chromatography, as described below.

### 2.3. Size Exclusion Chromatography

After removal of cells, the culture supernatant was concentrated to 500 µL by centrifugation through Amicon filter tubes at 1915× *g* in a Rotina 46 R centrifuge. OMVs were then purified by size exclusion chromatography using qEV exosome isolation columns from Izon Science, following the manufacturer’s instructions. Protein was detected in two elution peaks, comprising qEV fractions 7–9 and fractions 11–23. The purified OMVs (pooled fractions 8 and 9) were visualised by transmission electron microscopy (TEM) on a JEOL JEM1010 instrument after staining with 2% methylamine tungstate, as described by Kotrbová et al. [29]. The fluorescence of the OMVs was quantified spectrophotometrically at excitation and emission wavelengths of 575 nm and 620 nm, respectively, using a Hidex Sense instrument. The OMV preparations typically contained 20–25 mg of protein, as determined by Bradford’s assay [30], with specific fluorescence (RFU [relative fluorescence units] per µg of protein) values between 105 and 140 RFU/μg protein (3 times higher than for qEV fractions 11–23).

No degradation in the fluorescence was observed during the course of this study. However, due to the anticipated presence of proteolytic enzymes and as a general precautionary measure, batches of the OMV preparations were aliquoted immediately after purification and any aliquots which were not immediately tested were frozen at −80 °C until used.

### 2.4. OMV Fusion, OMV Killing and Cellular Predation Assays

Bacterial strains were grown to mid-exponential phase in 20 mL of LB (prey species) or DCY (*M. xanthus* strains), cells were sedimented by centrifugation, washed and resuspended in TM buffer (8 mM MgSO_4_, 10 mM Tris pH 7.8), with volumes adjusted to produce cell suspensions with the same optical density, at 600 nm (OD_600_) for each assay (roughly 0.9). Assays were performed in at least triplicate and Student’s *t*-test (two-tailed) was used to define whether the means were significantly different between treatments, with a *p*-value cut-off of 0.05, and Bonferroni corrections for multiple comparisons. Correlations were calculated via the least-squares method.

To assay OMV assimilation (most likely due to membrane fusion), recipient cell suspensions were incubated for 1 min at 30 °C, with fluorescent OMVs (100 µg protein) purified from vanillate-induced EH715 cultures. The cells were then re-sedimented, washed twice and resuspended in TM buffer, and their fluorescence was assayed at excitation and emission wavelengths of 575 nm and 620 nm, respectively. Control experiments omitted the prey cells and/or OMVs, and the fluorescence (RFU) of the control assays without added fluorescent OMVs was subtracted as background from the RFU with added OMVs. As the fluorescence (RFU) per µg of protein varied slightly for each batch of OMVs produced, apparent fusion (increase in RFU over control) was normalised against the RFU/µg protein of the OMV preparation and expressed as µg of OMV protein.

To measure OMV-mediated killing, the prey cell suspensions were incubated with and without OMVs extracted from EH715 (100 µg protein) for 60 min at 30 °C, in triplicate. The mixtures were then serially diluted, plated onto LB agar and incubated overnight. The resulting prey colonies were counted, and killing was expressed as the % killed relative to the ‘without-OMV’ controls. To ensure that normalising by OD_600_ for diverse prey species did not generate assays with substantially different numbers of cells, and therefore cell:OMV ratios, the cful/mL/OD values were assessed and found to only vary by a maximum factor of 1.29 between different prey species.

Assays of cellular predation involved spreading 100 µL of prey cell suspensions onto nutrient-free TM agar plates (TM buffer solidified with 1.5% w/v agar). After drying, plates were spotted in at least triplicate with 10 µL of a suspension of EH715, and incubated for 2 days at 30 °C, after which the growth of EH715 was recorded for each spot as the mean of the shortest and longest diameters.

### 2.5. Cloning of M. xanthus gapA and pgk Genes

The aLICator LIC cloning system (Thermo Fisher Scientific, Waltham, MA, USA) was used to clone the *M. xanthus* DK1622 *gapA* (glyceraldehyde-3-phosphate dehydrogenase) and *pgk* (phosphoglycerate kinase) coding sequences into pLATE52, which allows the expression of the cloned sequences as N-terminal 6His-tagged proteins (with a 28 residue N-terminal extension of sequence MAGSHHHHHHGMASMTGGQQMGRSGWEL).

Insert sequences were amplified by PCR (1 unit PCRBIO HiFi Taq polymerase, 1x PCRBIO buffer, 0.2 μM each of forward and reverse primers, 50 ng genomic DNA) with an initial denaturation at 94 °C for 30 s, 30 cycles (30 s at 94 °C, 30 s at 52 °C, 60 s at 68 °C), followed by a final extension at 68 °C for 5 min. The primer sequences (and their nucleotide locations in the *M. xanthus* DK1622 genome) were: ‘gapA Forward’: GGTTGGGAATTGCAAGCTACCCGGATTGC (nt 3,287,079), ‘gapA Reverse’: GGAGATGGGAAGTCATTAGACGCCCTTGGAGACG (nt 3,288,119), ‘pgk Forward’: GGTTGGGAATTGCAAATCCGTTACATCG (nt 3,288,129), and ‘pgk Reverse’: GGAGATGGGAAGTCATTACCGCGTCTCCAGCG (nt 3,289,346). PCR products were purified using the QIAquick PCR purification kit (Qiagen) and concentrated using ethanol precipitation: 0.1 volumes of 2 M sodium acetate (pH 5.2) were added followed by 2.5 volumes of ice-cold 100% ethanol, and incubated on ice for one hour. The sample was then centrifuged at 14,000× *g* for 30 min, the pellet washed twice with ice-cold 70% ethanol, air dried and resuspended in 10 µL 10 mM Tris-Cl (pH 8.5) buffer.

2 µL 5× LIC buffer was mixed with 1 µL T4 DNA polymerase and PCR-amplified DNA and incubated at room temperature for five minutes. 0.6 µL 0.5 M EDTA was then added, followed by 1 µL of the pLATE52 vector, and annealed for 10 min at room temperature. Calcium chloride competent *E. coli* TOP10 cells were made using a standard protocol [31] and transformed by introducing 3 µL of the annealed mixture and plating on LB agar containing 100 µg/mL ampicillin. Recombinant clones were assessed by colony PCR using LIC standard sequencing primers (‘LIC Forward’: TAATACGACTCACTATAGGG and ‘LIC Reverse’: GAGCGGATAACAATTTCACACAGG). The PCR conditions were as above, but with an extension temperature of 72 °C. PCR products were cleaned as before and sequenced on an ABI 3730 DNA Analyser.

### 2.6. Expression and Purification of 6His-GAPDH and 6His-PGK

Recombinant plasmids were introduced into One Shot BL21(DE3)STAR calcium chloride competent *E. coli* cells by transformation and plated on LB agar supplemented with ampicillin (100 µg/mL). The transformants were inoculated into LB medium supplemented with ampicillin and grown in a shaking incubator at 37 °C until an OD600 of 0.5–0.6 was reached, when cultures were induced with 1 mM IPTG and grown for a further three hours.

The induced cultures were centrifuged for 10 min at 16,800× *g*, resuspended in native purification buffer (50 mM NaH_2_PO_4_, pH 8.0 and 500 mM NaCl) and sonicated for two minutes (alternating 10 s on, 10 s off) at 30 W. The lysed cultures were centrifuged for 20 min at 10,700× *g*; the resulting supernatant was passed through a 0.45 µm filter and the protein concentration of each filtered supernatant was determined by a Bradford Assay [30].

Ni^2+^-nitrilotriacetic acid (Ni-NTA) purification columns (Thermo Fisher Scientific, Waltham, MA, USA) were washed with two column volumes (CVs) of binding buffer (300 mM NaCl, 10 mM imidazole and 50 mM NaH_2_PO_4_, pH 8.0). The filtered supernatant was added to a column and gently shaken at room temperature for 1 hr. The supernatant was then allowed to flow through the column and the column was then washed with 5 mL of wash buffer (as binding buffer but with 20 mM imidazole) three times. 6His-tagged protein was eluted from the column with three aliquots of 5 mL of elution buffer (as binding buffer but with 250 mM imidazole). 20 μL samples were taken at every stage of the purification for visualisation by SDS-PAGE.

The protein samples were visualised by SDS-PAGE. Samples were mixed with 2× loading buffer (100 mM Tris-HCl pH 6.8, 4% (w/v) SDS, 20% (w/v) glycerol, 0.2% (w/v) bromophenol blue, 200 mM DTT), boiled at 95 °C for five minutes and put on ice. Samples were loaded (20 μL per well) into 4% stacking and 15% resolving gels using a 40% acrylamide solution (Sigma-Aldrich, St. Louis, MO, USA, 37.5:1). An initial voltage of 75 V was applied for 10 min, followed by 150 V for one to two hours. The gels were fixed (50% ethanol, 10% acetic acid), stained (0.25% Coomassie Brilliant Blue G-250, 50% methanol and 10% acetic acid) and de-stained (30% methanol and 10% acetic acid).

Elution fractions containing pure recombinant protein were combined, concentrated to 2 mL and washed with 70 mL of imidazole-free native purification buffer to remove imidazole, using Amicon^®^ Ultra-15 10,000 molecular weight cut-off centrifugal concentrator tubes (Merck Millipore, Burlington, MA, USA). The identity of the purified protein was confirmed by electro-spray ionisation tandem mass spectrometry.

### 2.7. Enzymatic Assays of GAPDH Activity

The enzymatic activity of 6His-GAPDH was assayed using a modified version of the method of Zhang and Snyder [32], which monitors the production of NADH from NAD^+^, as glyceraldehyde-3-phosphate is converted into 1,3-bisphosphoglycerate. NAD^+^ and D-glyceraldehyde-3-phosphate were used in the assays at 10 mM and 5 mM, respectively, to ensure they were not limiting the rate of reaction. Production of NADH was monitored by measuring the absorbance at 340 nm using a Shimadzu UV-1800 spectrophotometer attached to a Peltier system set to 25 °C, and all of the solutions used were pre-warmed to 25 °C for 5 min. At time = 0, 6His-GAPDH was added to the assay mixture and the sample was cuvette blanked against an identical cuvette lacking 6His-GAPDH. Enzyme activity was calculated from the initial rate of absorbance change using an extinction coefficient for NADH at 340 nm of 6220 M^−1^cm^−1^.

### 2.8. Assays of Prey Cell Lysis and Prey Growth/Inhibition

The lysis capabilities of the OMVs and proteins were assessed using a molten agar-based assay [33]. Exponential phase *E. coli* were mixed with molten agar (20 mM Na_2_PO_4_, pH 7, 45 °C), poured into petri dishes and allowed to cool. 5 µL of test sample was spotted in triplicate onto the surface of the agar, and the plates were incubated overnight at 37 °C. The diameter (mm) of any zones of lysis was recorded.

To assess the impact of proteins and/or OMVs on the growth/inhibition of liquid cultured *E. coli*, OD_600_ was monitored using a clear-walled 96-well microplate, with measurements taken every 30 min for 20 h at 37 °C, with intermittent agitation at 120 rpm. In each well, 50 µL LB broth was mixed with 100 µL of the cells (OD_600_ = 0.2) and 100 µL of the test sample. The liquid culture growth/inhibition and cell lysis assays were repeated with increasingly dilute samples of the proteins and OMVs (10-fold dilutions).

## 3. Results

### 3.1. Inducing Expression of mCherry in M. xanthus Produces Fluorescent OMVs

**Figure 1:**
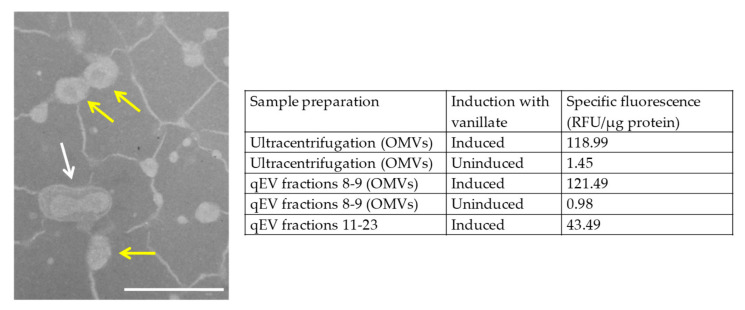
(**Left**): Transmission electron micrograph of OMVs purified from EH715 by size exclusion chromatography (pooled fractions 8 and 9). Example OMVs are shown with a yellow arrow, a potential fusion of two OMVs is indicated with a white arrow. Bar = 200 nm. (**Right**): Mean specific fluorescence values of samples prepared by qEV size exclusion chromatography and ultracentrifugation, induced and uninduced with 200 µM vanillate).

*M. xanthus* EH715 expresses mCherry from a vanillate-inducible promoter. Peak fluorescence of the EH715 cells was observed when supplementing the growth medium with 200 µM vanillate (Supplementary Figure S1). Previous studies of *M. xanthus* OMVs have only used ultracentrifugation, but for this study, we used both ultracentrifugation and size exclusion chromatography. Both methods produced OMVs with similar levels of specific fluorescence (mean values of 119 and 121 RFU/µg protein, respectively, compared to a mean of 1.2 RFU/µg protein for the OMVs prepared from the cultures which were not induced with vanillate). We were unable to image the OMVs by fluorescence microscopy; however, TEM imaging of the OMVs purified by size exclusion chromatography (Figure 1) revealed OMVs of the size expected for *M. xanthus* (around 50 µm diameter), connected by a network of filaments, possibly outer membrane tubes, dehydrated exopolysaccharide, or another artefact of sample preparation. In comparison, the OMVs produced by differential ultracentrifugation were less uniform in size, and appeared to contain contaminating aggregates, chains and tubes (Supplementary Figure S2). Although the OMV samples prepared by both methods gave similar results in our assays, the data below were acquired using batches of OMVs prepared by size exclusion chromatography. Supplementary Figure S2 and Figure 1 show that the samples prepared by centrifugation are not just OMVs, while size exclusion chromatography provided a sample containing predominantly OMVs. The size exclusion chromatography preparations are therefore reported as we can more confidently attribute any biological function to the OMVs, rather than a co-purifying contaminant.

### 3.2. Fluorescent OMVs Allow Quantification of Cargo Protein Delivery to Recipient Cells

**Figure 2:**
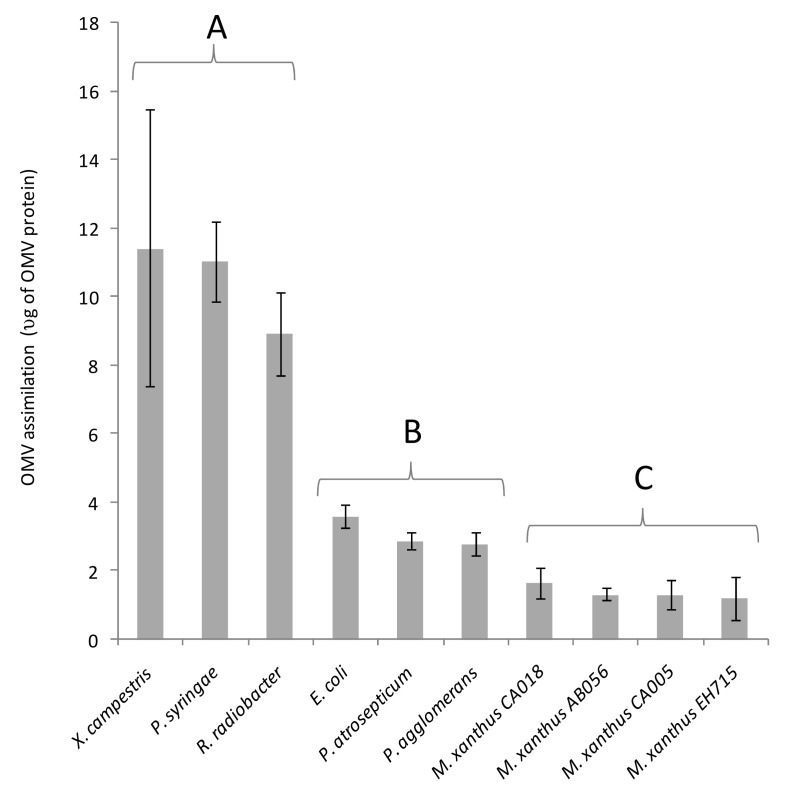
Assimilation of OMVs by a panel of Gram-negative bacteria. Error bars represent ± 1 standard deviation (*n* ≥ 3). A, B and C represent groups of organisms that exhibited substantially different OMV assimilation values from members of other groups. No statistically significant differences were observed within groups (Bonferroni corrected *p* < 0.05), but were observed between groups. Organisms are ordered by apparent fusion propensity.

It has been shown that the OMVs from a variety of organisms can fuse readily with the outer membranes of diverse Gram-negative bacteria [20,21,34,35]. As a proxy for fusion, the ability of different bacterial species to assimilate mCherry from *M. xanthus* OMVs was tested by incubating recipient cells with fluorescent OMVs purified from EH715. After incubation with OMVs, the recipient cells were re-isolated by centrifugation, washed thoroughly (to remove any loosely associated OMVs) and their fluorescence was measured using a Hidex Sense instrument; the background fluorescence from OMV-free controls was subtracted, and the increase in the fluorescence was normalised against the protein concentration of the OMV preparation (Figure 2). Recipient organisms tested included four strains of *M. xanthus* and six Gram-negative prey organisms. After mixing with fluorescent OMVs, all recipient cells increased in fluorescence significantly (*p* < 0.05).

Three prey recipients (*R. radiobacter, X. campestris* and *P. syringae*) acquired substantially more fluorescence than the other recipients tested (Figure 2). Apparent fusion of the OMVs was observed with all *M. xanthus* strains, however, it occurred at lower levels than for any of the non-myxobacterial recipient cells (mean OMV uptake was lowest for EH715, the OMV-producing strain). This suggests that re-fusion of OMVs with producing cells is somehow inhibited, and that inhibition of fusion is shared by phylogenetically similar organisms.

### 3.3. Cargo Protein Uptake Does Not Correlate with OMV Predatory Activity

**Figure 3:**
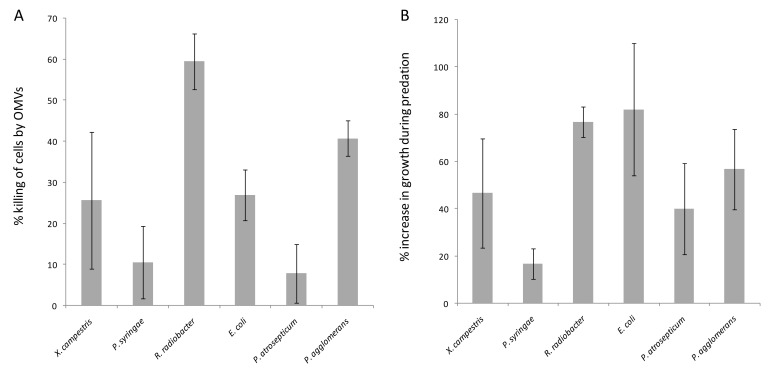
Predatory activity of isolated OMVs (**A**) and cells (**B**) of EH715. OMV killing is expressed as the % of prey cells killed by incubating with OMVs, while whole-cell killing is presented as the % increase in swarming growth when growing on prey (higher myxococcal growth means greater cellular predation). Error bars represent ± 1 standard deviation (*n* ≥ 3). Prey organisms presented in the same order as Figure 2.

The killing activity of purified OMVs was assayed for each prey organism. Prey cell suspensions were aliquoted and incubated with either OMVs or buffer. The numbers of surviving prey cells were assessed by dilution plating and counting colonies, and their susceptibility to predation was expressed as the % of prey cells killed by incubation with predator OMVs (Figure 3A). The percentage killing ranged between 8% (*P. atrosepticum*) and 60% (*R. radiobacter*).

For comparison, the predatory activity of EH715 cells was assayed by measuring colony expansion when grown on lawns of different prey organisms. Predatory activity is expressed as the percentage increase in the swarm diameter compared to the growth in the absence of prey and is shown in Figure 3B. While all prey stimulated swarm expansion, some prey only did so marginally (e.g., 17% increase on *P. syringae*), while other prey stimulated growth substantially (e.g., 77% increase on lawns of *R. radiobacter*).

The predatory activity of EH715 cells correlated strongly and positively with OMV killing activity (R = 0.71, *p* = 0.11), implying that prey susceptibility to predation is primarily due to their sensitivity to killing by OMVs, and consequently, that OMVs are the primary agents causing prey cell death during predation. However, OMV assimilation did not correlate with OMV-mediated killing (R = 0.03, *p* = 0.96), and correlated only weakly with the predatory activity of cells (R = 0.32, *p* = 0.54). Therefore, it seems that OMVs can kill prey organisms, regardless of how efficiently their OMVs might fuse with recipient cells. Calculating a ‘susceptibility ratio’ (percentage OMV-mediated killing divided by the amount of apparent OMV fusion) gives values ranging between 0.95 for *P. syringae* (which assimilates OMVs efficiently, but is highly resistant) and 14.6 for *P. agglomerans* (which assimilates little OMV material, yet is very sensitive to OMV-mediated killing). Presumably, reducing the efficiency of OMV fusion would not be an effective mechanism for predation resistance in prey, nor would increasing the fusion rate of OMVs be an effective strategy for predators to enhance their predatory activity.

### 3.4. Expression and Purification of Active M. xanthus 6His-GAPDH

In a previous publication, we presented evidence from killing assays in liquid medium, which suggested that a fusogenic enzyme, GAPDH, could stimulate the predatory activity of *M. xanthus* OMVs. Therefore, it was theorised that increased fusion of OMVs with prey cells would increase prey death [12]. However, a dichotomy emerges, as the results presented here instead suggest that an increased rate of fusion between OMVs and prey cells would not be expected to correlate with an increased OMV predatory activity. The experiment described by Evans et al. [12] was undertaken using commercially available GAPDH from *Oryctolagus cuniculus* (rabbit) and not *M. xanthus* GAPDH. Therefore, to resolve the apparent dichotomy, we made 6His-tagged *M. xanthus* GAPDH and tested its ability to stimulate OMV predatory activity. We also made *M. xanthus* 6His-tagged PGK (phosphoglycerate kinase). Both PGK and GAPDH are glycolytic enzymes and are encoded by the same operon in *M xanthus*. GAPDH is known to have membrane fusion activity, but PGK is not.

**Figure 4:**
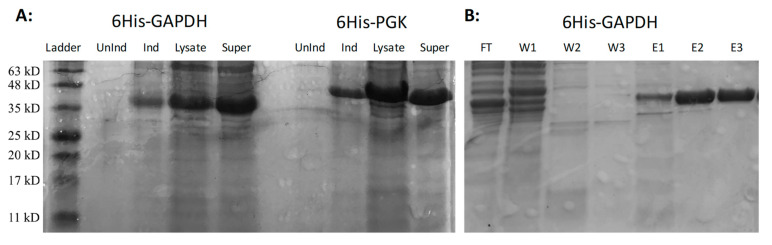
Expression and purification of fusion proteins. (**A**): 15% SDS-PAGE gel of purified 6His-GAPDH (left) and 6His-PGK (right). From left to right for each protein: uninduced cells, cells induced with 1 mM IPTG, sonicated cell lysate and the supernatant of centrifugated cell lysate. GAPDH and PGK proteins are the dominant bands at around 40 kDa and 46 kDa, respectively. (**B**): Ni^2+^-NTA purification of 6His-GAPDH. From left to right: Flow-through of unbound protein (FT), washes 1–3 (W1–W3) and elutions 1–3 (E1–E3).

The *M. xanthus pgk* (MXAN_2816) and *gapA* (MXAN_2815) genes, encoding PGK and GAPDH, respectively, were PCR-amplified and cloned into an expression vector, which allowed the inducible expression of 6His-tagged proteins in *E. coli*. On induction of expression, both fusion proteins were found to be soluble, with large quantities of protein in the supernatant of centrifuged cell lysates (Figure 4A). Purification using Ni^2+^-NTA columns resulted in pure preparations of fusion protein (Figure 4B).

To ensure 6His-GAPDH had adopted its native conformation, its glycolytic activity was assayed. An enzymatic assay measuring the production of NADH from NAD^+^ as glyceraldehyde-3-phosphate is converted to 1,3-bisphosphoglycerate, demonstrated that the purified 6His-GAPDH was active, with a specific activity of 3.9 units per mg of protein.

### 3.5. OMV-Dependent Lysis of Prey Is Not Stimulated by 6His-GAPDH in a Solid-Medium Based Assay

**Figure 5:**
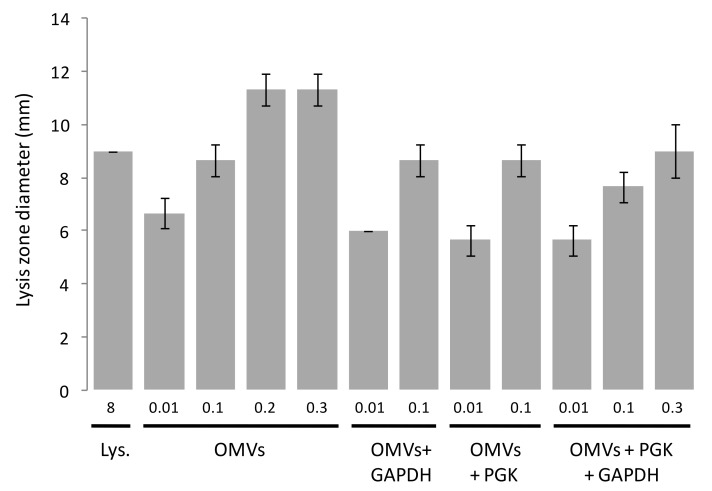
Lysis zones of GAPDH, PGK and OMVs on *E. coli* agar. Error bars reflect standard deviation (*n* = 3). Numbers below bars provide the concentration of each added component in mg/mL. Lys denotes lysozyme (positive control).

As an alternative to assaying the OMV killing of prey in liquid assays by measuring the reduction in prey cfu/mL, we employed a semi-quantitative solid medium assay which visualises the cellular lysis of prey bacteria. *E. coli* prey cells were embedded in agar and poured into petri dishes. Samples were then spotted in triplicate onto the surface of the plates and the zones of clearing were measured where prey cells had lysed (Figure 5).

No visible zones of clearing were observed when the purified 6His-GAPDH or 6His-PGK were tested on their own. However, visible zones were observed when the purified OMVs were tested, with the diameter of the zone of clearing increasing to a maximum as greater concentrations were added (Figure 5). Lysis of *E. coli* cells was not observed when adding OMVs at concentrations below 0.01 mg/mL. Adding 6His-GAPDH or 6His-PGK alongside OMVs did not increase the diameter of the zones of clearing, implying that 6His-GAPDH does not enhance the lytic activity of OMVs.

### 3.6. Native M. xanthus 6His-GAPDH and 6His-PGK Inhibit Prey Growth Independently of OMVs

**Figure 6:**
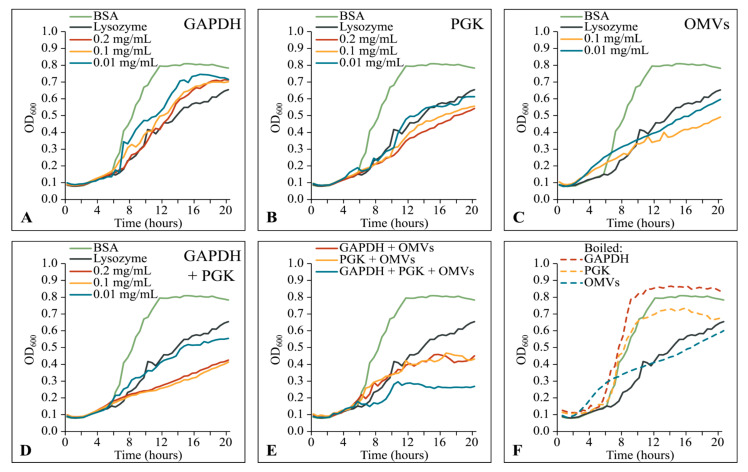
Growth curves of *E. coli* in the presence of GAPDH, PGK and/or OMVs. In graphs A-F BSA (bovine serum albumin) (0.1 mg/mL) and lysozyme (0.1 mg/mL) are shown in green and dark grey, respectively. Red, yellow and blue lines indicate 0.2, 0.1 and 0.01 mg/mL, respectively, of the added component in (**A**–**D**). (**A**) 6His-GAPDH. (**B**) 6His-PGK. (**C**) OMVs. (**D**) 6His-GAPDH and 6His-PGK. (**E**) OMV mixtures with enzymes (each component at 0.1 mg/mL). (**F**) 0.1 mg/mL of boiled 6His-GAPDH, 6His-PGK or OMVs. Lines represent the mean of three replicates.

We then assayed the effects of OMVs, 6His-GAPDH and/or 6His-PGK on the growth of *E. coli* by measuring the OD_600_ of cultures in a 96-well microplate every 20 min for 20 h (Figure 6). Over the course of the assays, *E. coli* supplemented with bovine serum albumin (as a negative control) grew steadily, reaching stationary phase and an OD_600_ of 0.8 after around 10 h. As a positive inhibitory control, adding lysozyme inhibited *E. coli* growth such that, even after 20 h, the OD_600_ of the culture had only reached around 0.6 (Figure 6A–F). OMVs at 0.01 or 0.1 mg/mL inhibited the growth of *E. coli* to a similar extent as adding 0.1 mg/mL lysozyme (Figure 6C).

Surprisingly, the addition of either 6His-GAPDH or 6His-PGK also inhibited the growth of *E. coli*, even in the absence of OMVs (Figure 6A,B). This inhibitory effect was additive, such that supplementing the culture with both 6His-GAPDH and 6His-PGK resulted in a substantially greater inhibition than that obtained with the lysozyme control (Figure 6D). In contrast with the inhibitory effects of OMVs, the inhibition observed on adding 6His-GAPDH or 6His-PGK was abolished if the enzymes were denatured before addition to the culture, indicating that their growth inhibiting activity requires a native folded enzyme (Figure 6F).

The addition of either 6His-GAPDH or 6His-PGK alongside OMVs did not increase the inhibitory activity of the OMVs substantially, however, adding 6His-GAPDH, 6His-PGK and OMVs all together gave the greatest level of inhibition observed. After an extended lag phase of around 9–10 h (compared to 5 h for the positive control), OD_600_ doubled from 0.15 to 0.3 over the course of 1–2 h, before growth completely ceased for the remainder of the assay (Figure 6E).

Our data show that 6His-GAPDH, 6His-PGK and OMVs from *M. xanthus* are independently inhibitory and have additive, rather than synergistic, effects on *E. coli* growth. Their currently unknown mechanism for prey growth inhibition/killing does not involve promoting fusion of OMVs with target cells, as was previously thought.

## 4. Discussion

The mechanism by which myxobacteria deliver toxins to prey is likely to differ depending on the species of prey organism. Against Gram-negative bacteria, myxobacterial OMVs can fuse with the prey outer membrane to deliver toxins directly into the periplasm [12]. This natural mechanism of OMV-mediated toxin delivery can be exploited to attack non-natural target organisms; for instance, OMVs can also fuse with the plasma membrane of eukaryotic cells, releasing toxins to attack intracellular Gram-positive pathogens [23,36,37]. Antibiotics are thought to be among the most toxic cargo molecules delivered to prey, however, purified antibiotics from myxobacteria tend to work more effectively against Gram-positive than Gram-negative bacteria [38,39,40]. Conversely, myxobacterial cells predate more efficiently on Gram-negative than on Gram-positive prey [4]. This apparent dichotomy is likely a consequence of the Gram-negative outer membrane, which protects the cell from exogenous antibiotics via various mechanisms [41], but also potentially provides an entry route for the intracellular delivery of OMV cargo molecules.

Nevertheless, Gram-positive bacteria and fungi can also be killed effectively by myxobacteria. OMVs can fuse with eukaryotic cells, and as Gram-positive bacteria can make extracellular vesicles, it seems likely that they might also be able to undergo fusion with OMVs [42]. However, OMVs can lyse at the surface of Gram-positive bacteria, releasing cargo molecules, and the composition of the extracellular milieu seems to be largely due to the lysis of secreted OMVs [12]. Thus, whether OMVs enter fungi and Gram-positive bacteria by fusion or not, OMVs are nevertheless likely to be important agents of prey cell killing during myxobacterial predation.

Although Myxobacteria are broadly toxic, some prey exhibit reduced sensitivity to myxobacterial attack. Some launch a counter-attack, some build fortified structures, while others possess de-toxification mechanisms [27,43,44,45,46]. As increasing OMV fusion is thought to stimulate the predatory activity of myxobacterial OMVs [12], we hypothesised that Gram-negative bacteria might also resist predation by preventing OMV fusion. To investigate the importance of fusion for predation, we therefore investigated the uptake of fluorescent OMVs by a range of Gram-negative prey.

When cultivated with vanillate to induce mCherry expression, the OMVs produced by *M. xanthus* EH715 were also fluorescent, and a similar size to those expressed by wild-type cells, whether prepared by ultracentrifugation or size exclusion chromatography (Figure 1, Supplementary Figure S2 and [12]). It would be interesting to additionally characterise the OMV samples using nanoparticle tracking, to provide a more quantitative measure of OMV production per cell and to assess the size distribution of OMVs. Unfortunately, we also cannot say whether the mCherry associated with OMVs is localised within the OMV lumen or bound to the OMV membrane. Wei et al. [47] fused a lipoprotein signal peptide to mCherry to visualise outer membrane tubes and outer membrane exchange of proteins in *M. xanthus*. Our mCherry construct did not contain a signal peptide and would therefore be expected to be cytoplasmic; nevertheless, mCherry is incorporated into the OMVs produced by EH715. Proteins predicted to be cytoplasmic are often found in OMVs, including those of *M. xanthus* [16,18], and it is believed that bacteria possess a mechanism for sorting cytoplasmic proteins into OMVs, although the nature of the sorting mechanism remains elusive [48].

We believe mCherry is associated with OMVs during their production, rather than being attached post-production, as the OMVs were purified from cultures which were not old enough to have undergone significant amounts of autolysis [49], making the post-production decoration of non-fluorescent OMVs with mCherry unlikely. We are also very confident that our OMV preps are specifically OMVs and not other types of vesicle or cellular debris. Proteomic analyses of the OMVs prepared by centrifugation have shown that the preps are highly statistically significantly enriched for proteins that have signal peptides, and 84% of those proteins in the OMV preps which can be assigned a cellular location are predicted to be outer membrane proteins or periplasmic [18].

On incubation with fluorescent OMVs, the fluorescence was incorporated into recipient cells, but to a different extent, depending on the recipient. EH715 and other strains of *M. xanthus* took up significantly less fluorescence than other recipients (Figure 2), implying that *M. xanthus* somehow inhibits the re-assimilation of *M. xanthus* OMVs. This contrasts with the process of outer membrane exchange, where the membranes of separate *M. xanthus* cells that are isogenic for the TraA membrane receptor can transiently fuse to allow the cell-cell exchange of membrane material [50].

The kinetics of membrane fusion are dependent on multiple variables, including membrane curvature, metal ions and lipid composition [51]. Lipid composition is particularly important as different lipids alter the curvature of membranes and the free energy change of membrane fusion [51]. In contrast to most bacteria, myxobacterial membranes contain rare, complex and varied lipids, and their membrane lipid profiles differ markedly between taxa [52,53,54]. Thus, it would be interesting to determine whether the OMV re-fusion inhibition mechanisms exist beyond the myxobacteria, and whether the OMV lipid composition is responsible for the inhibition of OMV re-fusion with *M. xanthus* cells.

As the amount of cargo protein uptake does not correlate with the killing activity of purified OMVs, it would appear that the potency of OMV cargo molecules (and/or whether the prey is resistant to them), rather than OMV fusion, dictates the sensitivity of prey to OMV-mediated killing. Nevertheless, there may be some conditions under which increased OMV fusion might increase predatory efficiency. For instance, in mixed populations, if prey organisms have similar resistance/susceptibility to OMV contents, those with a reduced propensity for OMV fusion might enjoy a selective advantage over competing organisms that are more prone to OMV fusion.

It is perhaps not overly surprising that the potency of cargo molecules is more important than the efficiency of OMV fusion. Li et al. [33] found that the predatory potency of OMVs from diverse organisms was dictated in part by the peptidoglycan (PG) chemotypes of the recipient organisms and of the OMV-producing strains. They argued that if the recipient had the same PG chemotype as that of the OMV-producer, the fusion of OMVs would allow PG hydrolases from the donor to break down the PG of the recipient, whereas having a different PG chemotype would make the recipient intrinsically resistant [33]. However, lysis was seen even when the donor/recipient chemotypes were very different, suggesting that further toxins, such as secondary metabolites and/or digestive enzymes such as lipases or proteases, were potentially involved.

Previously, we had shown that the addition of rabbit GAPDH increased the killing of prey cells by intact OMVs when assessed in liquid assays monitoring the prey cfu/mL. In the same assays, the rabbit GAPDH showed no predatory activity on its own, suggesting that it enhanced the activity of the OMVs. GAPDH is a well-characterised example of a moonlighting protein, a diverse class of proteins that have multiple independent activities [55]. As one of the known moonlighting activities of GAPDH is fusogenesis, we proposed that the rabbit GAPDH promoted killing by OMVs by increasing OMV fusion with the prey cells [12,56]. Surprisingly, our results above suggest that *M. xanthus* GAPDH does not have the fusogenic activity of rabbit GAPDH, as it cannot stimulate the lytic activity of OMVs. Even more surprisingly, our results demonstrate that *M. xanthus* 6His-GAPDH can inhibit the growth of *E. coli* in liquid culture. It is possible that rabbit GAPDH also has growth inhibitory activity against *E. coli*, as the assays reported by Evans et al. [12] with rabbit GAPDH measured reductions in the cfu/mL of the prey rather than growth rates. A growth-inhibitory moonlighting activity of full-length GAPDH has not been reported previously in the literature, although a small peptide derived from human GAPDH has been shown to inhibit the growth of *E. coli* [57].

As a negative control, PGK was chosen for investigation as it is encoded in the same operon as GAPDH, both enzymes are glycolytic, and both have numerous moonlighting activities [58]. However, to our knowledge, neither protein from any organism has been shown to possess bacteriostatic activity. While GAPDH has been found to be associated with OMVs and in soluble culture supernatants [17], PGK does not seem to be an OMV component, or even secreted alongside OMVs. The production of vesicles seems to be a ubiquitous property of membranes, and it is likely that the OMVs shed by ancestral myxobacteria were selected for their increased antimicrobial activity during the evolution of myxobacterial predatory activity. Perhaps the growth-inhibiting activity of secreted GAPDH also evolved uniquely in ancestral myxobacteria as they became predatory.

The results above confirm that the composition of the OMV cargo is crucial in dictating the predatory activity against prey organisms, more so than the OMV’s propensity to deliver cargo protein to target cells. Nevertheless, it would be interesting to investigate OMV-prey cell fusion further at the single cell level, to compare what happens in the well-mixed in vitro assays described here, to a more natural situation, with structured populations of predator and prey. Single cell/OMV studies would also be worthwhile to formally test the assumption that myxobacterial OMV cargo protein assimilation by prey occurs via membrane fusion, as has been observed for the OMVs of other organisms, or whether it occurs via some alternative mechanism.

We also need to identify which OMV cargo molecules are responsible for the specificity of predatory activity, and investigate their mechanisms of action. The ‘susceptibility ratio’ described above can provide an indication of which strains are most susceptible to particular OMVs, but could also be used to compare between OMVs from different predators to assess the relative potency of their cargo molecules. For instance, OMVs that exhibit the effective killing of a prey despite inefficient fusion would presumably contain a more potent cargo than OMVs from another predator that exhibited similar predatory activity but greater OMV fusion rates. A related aspect of cargo protein activity that is important to investigate (e.g., via protease digestion assays) is whether cargo proteins are internalised into recipient cells or absorbed onto the cell surface, and how that relates to the site of their predatory activity within prey cells.

Finally, it will also be interesting to further investigate the mechanism of the newly identified antimicrobial activity of GAPDH (and PGK). Potentially, GAPDH and PGK might promote predation by digesting outer membrane components or by hydrolysing the peptidoglycan of prey cells. Alternatively, they might cause the aggregation of prey cells, sterically hindering their ability to acquire nutrients and/or replicate efficiently. Both GAPDH and PGK are known to act as adhesins during bacterial pathogenesis [59,60,61], so a productive initial strategy might be to identify the molecular targets that each enzyme interacts with. It would also be worthwhile to investigate the structure of *M. xanthus* GAPDH and compare it to that of rabbit GAPDH to see whether structural differences could be related to their different activities. A multiple sequence alignment of GAPDH is provided in Supplementary Figure S3, which includes rabbit and *M. xanthus* GAPDH, and highlights conserved motifs. Whatever the mechanisms of GAPDH and PGK-mediated growth-inhibition might be, deciphering their modes of action can only help in understanding how myxobacteria kill their prey and how we can leverage that understanding to rationally exploit them as biological control agents.

## Data Availability

The data presented in this study are available on request from the corresponding author.

## Supplementary Materials

The following supporting information can be downloaded at: https://www.mdpi.com/article/10.3390/microorganisms11040874/s1, Figure S1: Fluorescence images of EH715 cells; Figure S2: Transmission electron micrograph of OMVs purified by differential centrifugation. Figure S3: Multiple sequence alignment of GAPDH.
