# Supplementary material for: Predatory Strategies of Myxococcus xanthus: Prey Susceptibility to OMVs and Moonlighting Enzymes
Source: Microorganisms. 2023 Mar 29;11(4):874. doi: 10.3390/microorganisms11040874 (PMC10141889; doi:10.3390/microorganisms11040874)
Supplement: Supplementary file 1 [file microorganisms-11-00874-s001.zip › Supplementary Figure S1.pdf]

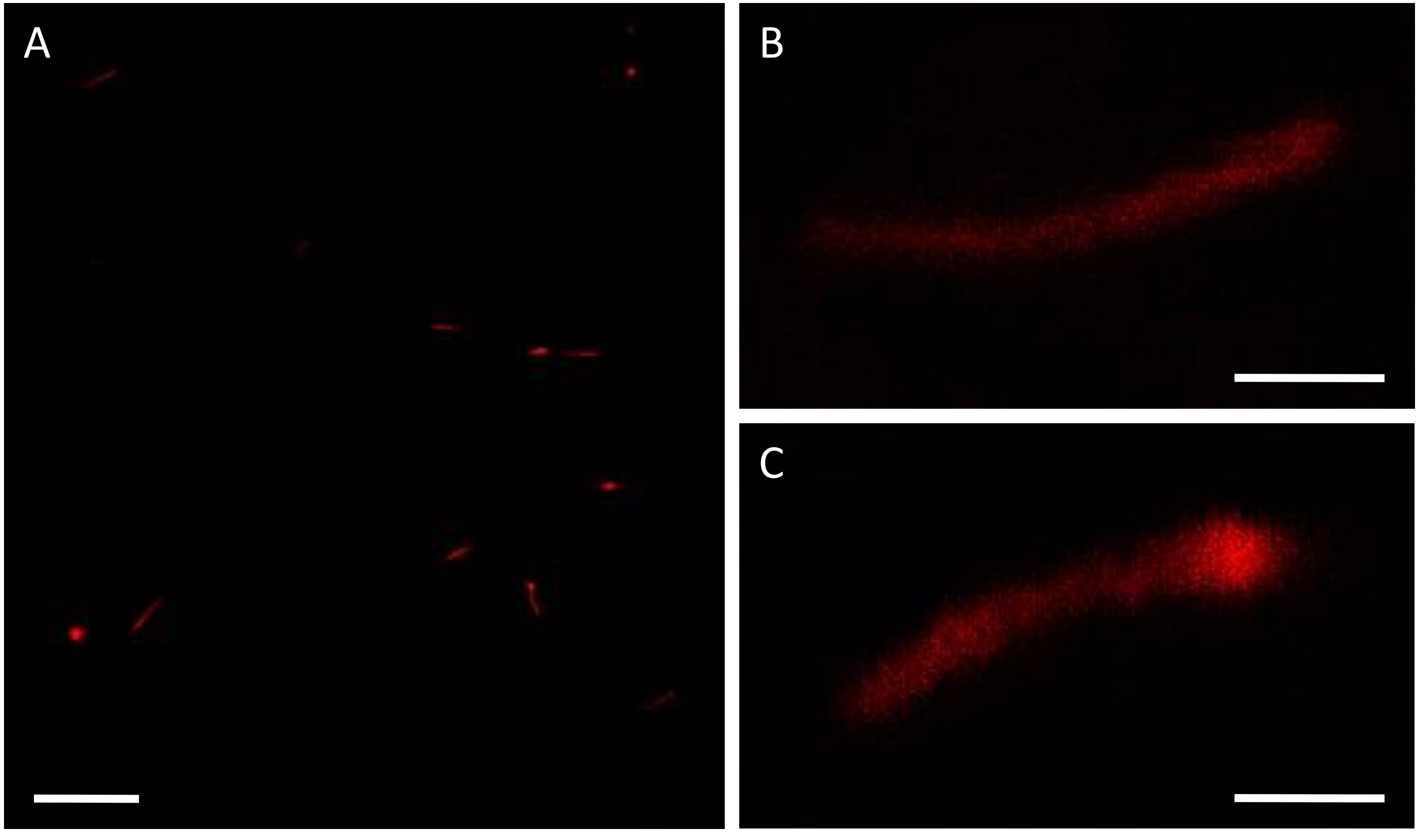

Supplementary Figure S1. Fluorescence images of EH715 cells. A-C: Laser confocal microscope images of EH715 cells expressing mCherry (excitation at 560 nm, emission at 620 nm). Bar is 20 µm in panel A and 2 µm in panels B and C.
