# Supplementary material for: Predatory Strategies of Myxococcus xanthus: Prey Susceptibility to OMVs and Moonlighting Enzymes
Source: Microorganisms. 2023 Mar 29;11(4):874. doi: 10.3390/microorganisms11040874 (PMC10141889; doi:10.3390/microorganisms11040874)
Supplement: Supplementary file 1 [file microorganisms-11-00874-s001.zip › Supplementary Figure S2.pdf]

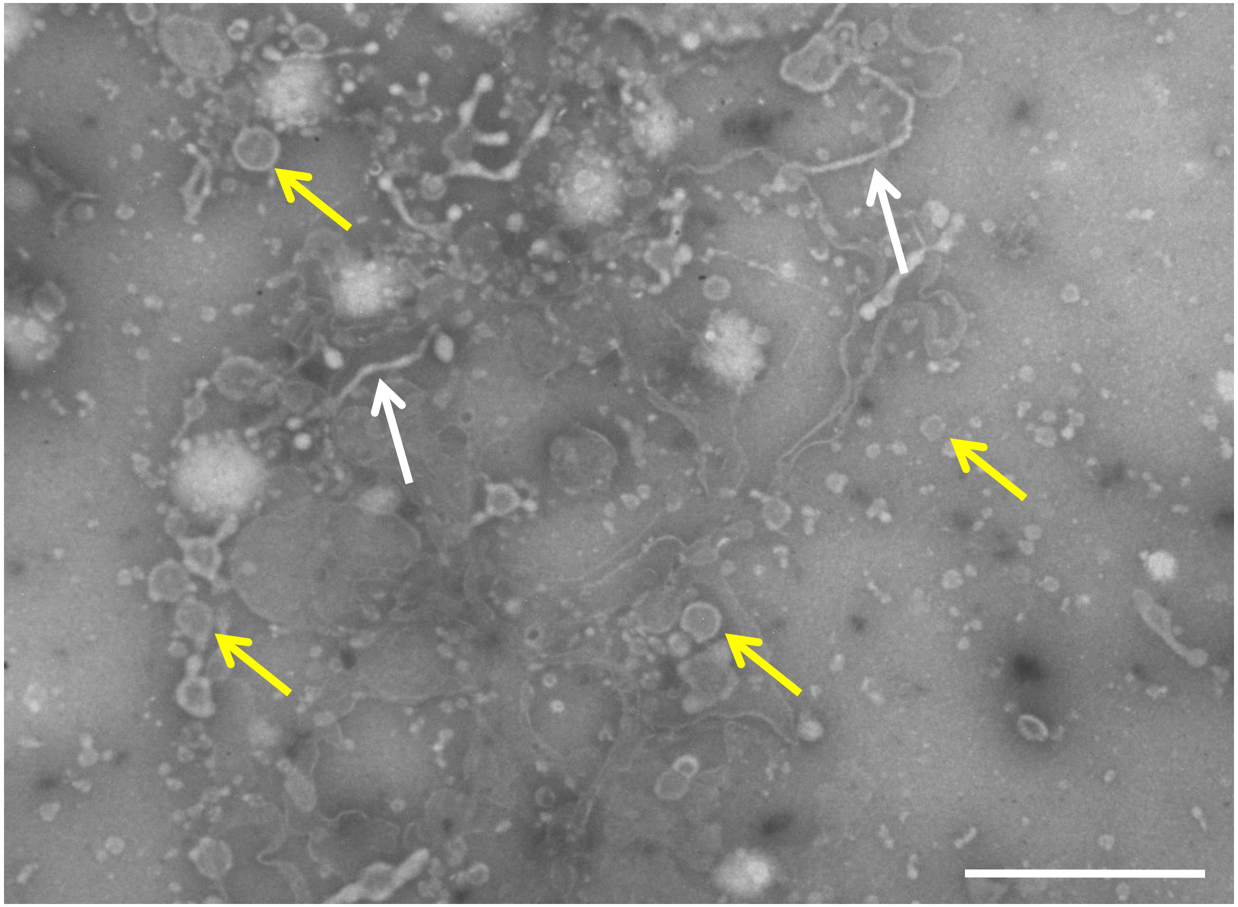

Supplementary Figure S2. Transmission electron micrograph of OMVs purified by differential centrifugation. Example OMVs are shown with yellow arrows. Linear features (white arrows) are possibly outer membrane chains or tubes. Bar = 1 μm.
