# Supplementary material for: Predatory Strategies of Myxococcus xanthus: Prey Susceptibility to OMVs and Moonlighting Enzymes
Source: Microorganisms. 2023 Mar 29;11(4):874. doi: 10.3390/microorganisms11040874 (PMC10141889; doi:10.3390/microorganisms11040874)
Supplement: Supplementary file 1 [file microorganisms-11-00874-s001.zip › Supplementary Figure S3.pdf]

```
                                  10         20         30         40         50         60         70         80         90        100        110        120
                         ....|....| ....|....| ....|....| ....|....| ....|....| ....|....| ....|....| ....|....| ....|....| ....|....| ....|....| ....|....| ....|....|
Bacillus subtilis        -MKVKVAING FGRIGRMVFR KAMLD-DQIQ VVAINA-SYS AETLAHLIKY DTIHGRYDKE VVAGEDSLIV NGKKVLLLNS RDPKQLPWRE YDIDIVVEAT GKFNAKDKAM GHIEAGAKKV
Pseudomonas aeruginosa   -MTIKVGING FGRIGRIVFR AAQKR-SDIE IVAIND-LLD ADYMAYMLKY DSTHGRFDGT VEVKDGHLIV NGKKIRVTAE RDPANLKWDE VGVDVVAEAT GLFLTDETAR KHITAGAKKV
Escherichia coli         --MSKVGING FGRIGRLVLR RLLEVKSNID VVAIND-LTS PKILAYLLKH DSNYGPFPWS VDFTEDSLIV DGKSIAIYAE KEAKNIPWKA KGAEIIVECT GFYTSADKSQ AHLDAGAKKV
Myxococcus xanthus       -MATRIAING FGRIGRCILR AVLSRKEDLE IVAIND-LDK PAALAHLFKY DSVHRTWPGE VKATEKGIVV DGKEITVTAE KDPTALPWKS LNVDVVLECT GRFTARDAAE KHLKAGAKKV
Oryctolagus cuniculus    --MVKVGVNG FGRIGRLVTR AAFNS-GKVD VVAINDPFID LHYMVYMFQY DSTHGKFHGT VKAENGKLVI NGKAITIFQE RDPANIKWGD AGAEYVVEST GVFTTMEKAG AHLKGGAKRV
Homo sapiens             MGKVKVGVNG FGRIGRLVTR AAFNS-GKVD IVAINDPFID LNYMVYMFQY DSTHGKFHGT VKAENGKLVI NGNPITIFQE RDPSKIKWGD AGAEYVVEST GVFTTMEKAG AHLQGGAKRV
Gallus gallus            --MVKVGVNG FGRIGRLVTR AAVLS-GKVQ VVAINDPFID LNYMVYMFKY DSTHGHFKGT VKAENGKLVI NGHAITIFQE RDPSNIKWAD AGAEYVVEST GVFTTMEKAG AHLKGGAKRV
Candida albicans         -MAIKIGING FGRIGRLVLR VALGR-KDIE VVAVNDPFIA PDYAAYMFKY DSTHGRYKGE VTASGDDLVI DGHKIKVFQE RDPANIPWGK SGVDYVIEST GVFTKLEGAQ KHIDAGAKKV

                                 130        140        150        160        170        180        190        200        210        220        230        240
                         ....|....| ....|....| ....|....| ....|....| ....|....| ....|....| ....|....| ....|....| ....|....| ....|....| ....|....| ....|....| ....|....|
Bacillus subtilis        ILTAPGKNED VTIVMGVNED QFDAERHVII SNASCTTNCL APVVKVLDEE FGIESGLMTT VHAYTNDQKN IDNP-HKDLR RARACGESII PTTTGAAKAL SLVLPHLKGK LHGLALRVPV
Pseudomonas aeruginosa   VMTGPSKDNT PMFVKGANFD KYAG--QDIV SNASCTTNCL APLAKVINDN FGIIEGLMTT VHATTATQKT VDGPSHKDWR GGRGASQNII PSSTGAAKAV GKVLPELNGK LTGMAFRVPT
Escherichia coli         LISAPAGE-M KTIVYNVNDD TLDGN-DTIV SVASCTTNCL APMAKALHDS FGIEVGTMTT IHAYTGTQSL VDGPRGKDLR ASRAAAENII PHTTGAAKAI GLVIPELSGK LKGHAQRVPV
Myxococcus xanthus       IISAPAKGPD ITIAYGINHN EYDPAKHHVI SNASCTTNCL APIAKVLVDN FGIEKGLMTT VHSYTNDQRI LDLT-HEDMR RARAAALSMI PTSTGAAKAI GEVIPQLKGK MHGLAVRVPT
Oryctolagus cuniculus    IISAPSXD-A PMFVMGVNHE KYDNS-LKIV SNASCTTNCL APLAKVIHDH FGIVEGLMTT VHAITATQKT VDGPSGKLWR DGRGAAQNII PASTGAAKAV GKVIPELNGK LTGMAFRVPT
Homo sapiens             IISAPSAD-A PMFVMGVNHE KYDNS-LKII SNASCTTNCL APLAKVIHDN FGIVEGLMTT VHAITATQKT VDGPSGKLWR DGRGALQNII PASTGAAKAV GKVIPELNGK LTGMAFRVPT
Gallus gallus            IISAPSAD-A PMFVMGVNHE KYDKS-LKIV SNASCTTNCL APLAKVIHDN FGIVEGLMTT VHAITATQKT VDGPSGKLWR DGRGAAQNII PASTGAAKAV GKVIPELNGK LTGMAFRVPT
Candida albicans         IITAPSAD-A PMFVVGVNED KYTPD-LKII SNASCTTNCL APLAKVVNDT FGIEEGLMTT VHSITATQKT VDGPSHKDWR GGRTASGNII PSSTGAAKAV GKVIPELNGK LTGMSLRVPT

                                 250        260        270        280        290        300        310        320        330        340
                         ....|....| ....|....| ....|....| ....|....| ....|....| ....|....| ....|....| ....|....| ....|....| ....|....| ....|....| ....|..
Bacillus subtilis        PNVSLVDLVV DLKTDVTAEE VNEAFKRAAK TSMYGVLDYS DEPLVSTDYN TNPHSAVIDG LTTMVMED-- -RKVKVLAWY DNEWGYSCRV VDLIRHVAAR MKHPSAV
Pseudomonas aeruginosa   PNVSVVDLTV RLEKAATYEQ IKAAVKAAAE GEMKGVLGYT EDDVVSTDFN GEVCTSVFDA KAGIALND-- -NFVKLVSWY DNETGYSNKV LDLIAHISK- -------
Escherichia coli         KTGSVTELVS ILGKKVTAEE VNNALKQATT N--NESFGYT DEEIVSSDII GSHFGSVFDA TQTEITAVGD LQLVKTVAWY DNEYGFVTQL IRTLEKFAKL -------
Myxococcus xanthus       PNVSLVDLTV NTEKKVTAEA VIEAFRKAAE GPLKGVLEFS DAQTVSVDYN GNPHSAIFDS TNCFVMGD-- -NLLKVMAWY DNEWGFSNRM VDTAKFLVSK GV-----
Oryctolagus cuniculus    PNVSVVDLTC RLEKAAKYDD IKKVVKQASE GPLKGILGYT EDQVVSCDFN SATHSSTFDA GAGIALND-- -HFVKLISWY DNEFGYSNRV VDLMVHMASK E------
Homo sapiens             ANVSVVDLTC RLEKPAKYDD IKKVVKQASE GPLKGILGYT EHQVVSSDFN SDTHSSTFDA GAGIALND-- -HFVKLISWY DNEFGYSNRV VDLMAHMASK E------
Gallus gallus            PNVSVVDLTC RLEKPAKYDD IKRVVKAAAD GPLKGILGYT EDQVVSCDFN GDSHSSTFDA GAGIALND-- -HFVKLVSWY DNEFGYSNRV VDLMVHMASK E------
Candida albicans         TDVSVVDLTV RLKKAASYEE IAQAIKKASE GPLKGVLGYT EDAVVSTDFL GSSYSSIFDE KAGILLSP-- -TFVKLISWY DNEYGYSTRV VDLLEHVAKA SA-----
```

Supplementary Figure S3. Alignment of GAPDH sequences from a variety of prokaryotes and eukaryotes. Columns are shaded if residues have conservation identity (black) or similarity (grey) exceeding 85% according to the BLOSUM 62 substitution matrix.
